# Secretome from human placenta-derived mesenchymal stem cells repairs mechanically induced meniscus injury in mice by activating the proliferation and suppressing the apoptosis of endogenous meniscus progenitor cells

**DOI:** 10.1186/s13287-025-04688-6

**Published:** 2025-10-14

**Authors:** Wei-Heng Chen, Wei-Yu Lai, Duy-Cuong Le, Jui-Chien Hsing, Mai-Huong T. Ngo, Cheng-Xiang Kao, Kang-Yun Fan, Gee-Way Lin, Thai-Yen Ling, Yung-Che Kuo, Yen-Hua Huang

**Affiliations:** 1 Department of Biochemistry and Molecular Cell Biology, School of Medicine, College of Medicine, Taipei Medical University, Taipei, 11031 Taiwan; 2 School of Medicine, College of Medicine, Taipei Medical University, Taipei, 11031 Taiwan; 3 International Ph.D. Program in Cell Therapy and Regenerative Medicine, College of Medicine, Taipei Medical University, Taipei, 11031 Taiwan; 4 Vinmec Phu Quoc International Hospital, Bai Dai Area, Phu Quoc Special Zone, An Giang, 92551 Vietnam; 5 Graduate Institute of Medical Sciences, College of Medicine, Taipei Medical University, Taipei, 11031 Taiwan; 6 Department of Pathology, Keck School of Medicine, University of Southern California, Los Angeles, CA 90033 USA; 7 Department and Graduate Institute of Pharmacology, College of Medicine, National Taiwan University, Taipei, 10617 Taiwan; 8 TMU Research Center for Cell Therapy and Regeneration Medicine, Taipei Medical University, Taipei, 11031 Taiwan; 9 Core Laboratory of Good Tissue Practice, Office of Research and Development, Taipei Medical University, Taipei, 11031 Taiwan; 10 Center for Reproductive Medicine, Taipei Medical University Hospital, Taipei Medical University, Taipei, 11031 Taiwan

**Keywords:** Meniscus injury, Cellular therapy, Mesenchymal stem cells, Secretome, Meniscus progenitor cells, Meniscus regeneration, Exosomal miRNA

## Abstract

**Background:**

Meniscus diseases present certain therapeutic limitations. Although meniscectomy is the primary treatment option for meniscus injury (MI), this approach may accelerate the development of osteoarthritis and other degenerative joint diseases, and its therapeutic efficacy remains controversial. While human mesenchymal stem cells (MSCs) have emerged as a promising treatment option for MI, particularly in promoting cell proliferation and preventing apoptosis, their effect on activating endogenous meniscus progenitor cells (MPCs) to ameliorate MI and the underlying mechanisms remain unclear.

**Methods:**

The secretome was collected from human placenta-derived MSCs (pcMSCs). A cellular model of MI was established by challenging mouse MPCs with H_2_O_2_. Male C57BL/6 mouse model of MI was established by mechanically destabilizing the medial meniscus (DMM). Protein expression was analyzed through Western blotting, flow cytometry, and immunohistochemistry staining. After secretome administration, behavioral activity was assessed through gait analysis and rotarod tests. Key secretome factors were identified through cytokine arrays and microRNA (miRNA) analysis.

**Results:**

The pcMSC secretome significantly mitigated MI in both cellular and mouse models, as indicated by gait analysis (*P* < 0.05), rotarod tests (*P* < 0.01), histological analysis (safranin-O staining, *P* < 0.001), and immunohistochemical staining for apoptosis marker (Caspase-3) and MPC proliferation markers (Gli-1, Sca-1, and Ki67). Cytokine arrays revealed several factors associated with immunomodulation (MCP1 and MCP3), regeneration and angiogenesis (IGF-1, ANG, and VEGFA), osteogenesis (OPG and OPN), and extracellular matrix preservation (TIMP1 and TIMP2). Furthermore, exosomal miRNA analysis revealed target genes involved in endogenous stem cell activation (*SUFU* and *RUNX2*), apoptosis regulation (*Caspase-3*), anti-inflammatory responses (*IL-1β*, *IL-6*, and *PTEN*), ECM formation (*TRAF6* and *MMPs*), anti-cartilage matrix degradation (*mTOR*, *AKT2*, *AKT3*,* and COL10A1*), and cell migration (*ADAM family*).

**Conclusions:**

To the best of our knowledge, this is the first study demonstrating that the human pcMSC secretome promotes meniscus regeneration through activating endogenous meniscus progenitor cells both in vivo and in vitro. Our findings suggest that these regenerative effects are mediated by growth factors and exosomal miRNAs in the pcMSC secretome. The potential exosomal miRNAs effectively modulated ECM formation, anti-apoptosis, anti-inflammation, and anti-cartilage matrix degradation to mitigate MPCs injury. Overall, this study provides valuable insights into potential stem cell-derived secretome cell-free therapies for patients with exercise-induced meniscus injuries.

**Supplementary Information:**

The online version contains supplementary material available at 10.1186/s13287-025-04688-6.

## Background

Meniscus injury (MI), which commonly affects athletes and active individuals, is characterized by damage to the crescent-shaped cartilage in the knee that cushions the femur and tibia bones. The incidence of MI is approximately 6‰–7‰ [1]. Evidence suggests that MI accounts for a substantial proportion of all cases of knee injury [2]. MI typically manifests as pain, swelling, and limited mobility, which markedly impair activities of daily living. Degenerative meniscus lesions can develop gradually and progress to osteoarthritis.

Managing MI is challenging because of the interpatient variations in injury severity and tear location. Conservative treatment approaches, such as rest, ice application, compression, and physical therapy, may be effective for minor MI, but severe cases often necessitate surgical intervention. The meniscus’s limited blood supply hinders healing and recovery, complicating treatment. Furthermore, concurrent damage such as ligament tears can complicate treatment plans and extend rehabilitation, necessitating personalized therapy.

Despite its limitations, surgery has traditionally been considered the primary treatment option for individuals with untreatable MI. Surgical removal of the meniscus is associated with a poor prognosis, including the development of osteoarthritis, raising concerns regarding the long-term benefits of surgery. Meniscus sutures and repairs may fail to heal appropriately and often necessitate secondary surgery owing to inadequate blood supply. Moreover, meniscus allograft transplantation carries the risks of immune rejection and disease transmission. Recent randomized controlled trial has demonstrated no significant difference in efficacy between meniscal surgery and conservative treatment approaches [3]. Given that suturing is not feasible for severe MI and that meniscectomy may not be the optimal therapeutic approach for this injury, novel therapeutic strategies are urgently required.

Recently, cell therapy has emerged as a promising alternative for the treatment of MI [4]. Among various approaches of cell therapy, mesenchymal stem cells (MSCs) have demonstrated major clinical potential. MSCs derived from bone marrow [5], adipose tissue [6], synovium [7], and meniscus tissue [8] have been used to promote meniscal regeneration and prevent osteoarthritis [9]. Each MSC source has its distinct advantages and disadvantages. Bone marrow-derived MSCs (BMMSCs) enhance extracellular matrix (ECM) production and fibrocartilage-like tissue formation, facilitating meniscal regeneration in the avascular zone [10, 11]. Synovial MSCs (SMSCs) exhibit a gene expression profile that is more similar to the gene expression profile of meniscal cells than to that of BMMSCs [12]. SMSCs also exhibit superior chondrogenic and repair capabilities. Adipose–derived MSCs have gained attention because of their accessibility and abundance; these MSCs exhibit enhanced chondrogenic and differentiation potential [6].

MSC–conditioned medium (CM), also known as the secretome, has been demonstrated to promote cartilage formation and enhance the chondrogenic phenotype [13]. Compared with MSCs, MSC–CM can easily penetrate smaller wounds and exert lasting effects in vivo [14]. In addition, through its immunomodulatory properties, MSC–CM can alleviate joint inflammation and accelerate wound healing.

In addition to MSCs, meniscus progenitor cells (MPCs) play a crucial role in meniscus regeneration following injury, holding promise as an alternative therapeutic option [15]. These cells possess self-renewal capacity, multidifferentiation potential, and immunosuppressive properties. Studies on MPC-based meniscus tissue engineering [8, 13, 16] have indicated that MPCs have substantial regenerative potential and can delay or prevent osteoarthritis progression following meniscectomy. Located in the meniscus’s superficial zone, MPCs exhibit stem cell characteristics and express specific markers (e.g., CD44 and Sca-1) and genes associated with cartilage and collagen formation.

Although studies have highlighted several potential treatment options for MI, the precise mechanisms underlying meniscal repair remain largely unknown. We previously developed a serum-free selection system for cultivating human placenta-derived MSCs (pcMSCs) [17]. Evidence suggests that pcMSCs exhibit strong immunomodulatory and regenerative properties, underscoring their potential for treating inflammation-related conditions [17–24]. Chen et al. [20] reported that pcMSCs effectively suppressed hyperinflammatory responses in patients with COVID-19-associated acute respiratory distress syndrome, preventing 28-day mortality in the treatment group. Notably, pcMSCs have been approved by the US Food and Drug Administration for a Phase I clinical trial (NCT05886985) [25], which confirms the preliminary safety of pcMSCs and their derivatives for clinical use. In addition to pcMSCs, our previous studies have shown that exosomes derived from pcMSCs alleviate oxidative stress, inflammation, and mitochondrial injury, mitigating lung and liver damage in endotoxin-treated obese mice [19, 21].

In the present study, we explored the therapeutic potential of the human pcMSC secretome for MI, given its immunomodulatory, anti-apoptosis, cartilage matrix preservation, ECM formation, and MPC-proliferative properties. These properties were analyzed both in vivo and in vitro. For the in vivo experiment, we established a mouse model of MI through mechanically destabilizing the medial meniscus (DMM) surgery. For the in vitro experiment, we established a cellular model of MI by challenging mouse MPCs with H_2_O_2_. The results indicated that the pcMSC secretome promoted meniscus repair by activating the proliferation and suppressing the apoptosis of MPCs. Additionally, we explored key exosomal miRNAs and their target genes and signaling cascades involved in meniscus repair. To the best of our knowledge, this is the first study to elucidate the mechanisms underlying the pcMSC secretome–mediated repair of MI. Our findings may inform effective stem cell therapies for MI.

## Materials and methods

### MPC culture

MPCs were isolated from C57BL/6 mice. Briefly, C57BL/6 mice were euthanized by cervical dislocation under anesthesia with 2%–5% isoflurane (Panion & BF Biotech Inc., Taiwan) via inhalation. The knee joints were dissected using scissors and tweezers. After careful excision of the meniscus, the tissue was rinsed three times with phosphate-buffered saline (PBS) and then with 75% ethanol to minimize contamination risks. Under sterile conditions, the meniscus tissue was finely minced using microscissors and then digested with collagenase for 6 h. After digestion, the meniscus fragments were placed on a 12-well culture plate. The MPCs were maintained in 1.5 mL of growth medium, which comprised Minimum Essential Medium Alpha (Life Technologies, Rockville, MD, USA), 10% fetal bovine serum (Life Technologies), and 1% penicillin/streptomycin (Life Technologies).

### Cell viability assay

To assess cell viability, meniscal cells were first counted and then seeded (3000, 4000, or 5000 cells/well) onto a 96-well culture plate. The cells were cultured at 37 °C for 12 h in the presence of 5% CO_2_. The following time points were set for viability assessment: 0, 24, 48, and 72 h. WST reagent (Dojindo, Kumamoto, Japan) was added to each well 3 h before assessment. After incubation with the reagent at 37 °C for 3 h, optical density was measured at 450 and 600 nm on a microplate reader (Tecan, Männedorf, Switzerland).

### Flow cytometry

MPCs were resuspended in stain buffer (BD Pharmingen, San diego, CA, USA) and then incubated for 1 h with various antibodies—phycoerythrin (PE)–conjugated antibodies against CD34 or CD44, Alexa 647–conjugated antibodies against Sca-1 or CD140, or fluorescein isothiocyanate (FITC)–conjugated antibodies against CD105. After incubation, the cells were analyzed using a BD FACSVerse Cytometer (BD Biosciences). The antibodies and experimental conditions are presented in Supplementary Table S1.

### Extraction of the human PcMSC secretome

The pcMSC secretome was sourced from Professor Thai-Yen Ling’s laboratory (National Taiwan University, Taiwan), and collected as described previously [18]. Briefly, pcMSCs were seeded onto 10-cm culture plates (density: 5 × 10⁵ cells/plate) and cultured in serum-free MCDB201 medium. After 24 h of cell adhesion, the plates were washed three times with PBS and incubated in fresh serum-free medium for an additional 48 h to obtain the pcMSC–CM.

The secretome of pcMSC–CM was harvested through centrifugation at 3000 ×*g* for 10 min at 4 °C. The supernatant was collected and filtered through a 0.22 μm polyethylene terephthalate filter. After filtration, the secretome was stored at − 80 °C for future use. To prevent the degradation of the secretome’s paracrine factors, each storage tube was carefully handled, ensuring only one freeze–thaw cycle.

### Establishment of a cellular model of MI

To establish a cellular model of MI, mouse MPCs were cultured to 50%–60% confluence. Then, the cells were exposed to H_2_O_2_ at concentrations of 0, 100, or 200 µM for either 6–24 h to induce apoptosis and senescence. After this H_2_O_2_ challenge, the MPCs were divided into three groups: a blank group (supplemented only with serum-free MCDB201 medium), a mock group (supplemented with serum-free MCDB201 medium, epidermal growth factor, and insulin–transferrin–selenium solution), and a secretome group (supplemented with the human pcMSC secretome). After 7 days, the MPCs were harvested to evaluate apoptosis and proliferation markers. Uninjured mouse MPCs cultured in standard medium were used as an additional control. Cell viability was assessed using a WST assay.

### Establishment of a mouse model of MI

To establish a mouse model of MI, 7-week-old male C57BL/6 mice were obtained from the National Laboratory Animal Center, Taipei, Taiwan. The mice were housed at the laboratory animal center of Taipei Medical University under conditions of 22 ± 2 °C, 55% ± 10% humidity, and a 12-h light–dark cycle. They had free access to a standard diet and water. All mice were subjected to 2 weeks of pretraining to acclimate them to rotarod and gait tests (week − 2). After training, MI was induced through DMM surgery (week 0), which was performed under appropriate anesthesia with 2%–5% isoflurane via inhalation, as described previously [26]. Mice were included in this study if we observing the anterior half of the medial meniscus in the left leg was successfully resected. The wound was carefully sutured, and analgesia was administered to the mice for three continuous days (2 mg/kg of ketoprofen, subcutaneous injection, dosing every 24 h). Given that this is a pilot study to evaluate the healing effect of pcMSC-secretome on meniscus injury, six mice with MI (week 0) were randomly allocated to two cages. After 2 weeks, these two cages were randomly assigned as a PBS group (control, treated with PBS, *n* = 3) and a secretome group (treated with the human pcMSC-secretome, *n* = 3). PBS (15 µL) or the pcMSC secretome (23.85 µg in 15 µL) was injected into the knee joint of each mouse. Finally, motor function was evaluated weekly through rotarod and gait tests until week 11 in the animal center; the machine was also carefully cleaned after each mouse completed the experiment to minimize the odor effect of previously tested mice. After treatment, the mice did not show any signs of adverse events. In this study, all six mice did not meet the exclusion criteria of human endpoints. At week 12, the mice were euthanized by cervical dislocation under anesthesia with 2%–5% isoflurane via inhalation, and specimens were collected to evaluate meniscus regeneration. The work has been reported in line with the ARRIVE guidelines 2.0.

### Histological analysis and Immunofluorescence staining

For histological analysis, mouse meniscus tissue samples were decalcified, dehydrated, and embedded in paraffin. Next, the embedded samples were sectioned into 3-µm-thick slices and subjected to hematoxylin and eosin (H&E) and safranin-O (SO) staining. Finally, the stained sections were examined and photographed using a BX51 microscope (Olympus, Tokyo, Japan).

For immunofluorescence staining, the paraffin-embedded tissue sections (thickness: 3 μm) were incubated at 60 °C for 30 min and then deparaffinized in xylene (two baths, 10 min each). Next, the sections were rehydrated through incubation in a series of graded alcohol (100% twice, 95%, 80%, 70%, and 50%) and distilled water, with each bath lasting 3 min. Relevant antigens were retrieved by incubating the deparaffinized sections in 10 mM citrate buffer (pH 6.0) at 98 °C for 10 min, followed by cooling at room temperature for 1 h. Subsequently, the tissue sections were washed with Tris-buffered saline (TBS) and permeabilized using TBS containing 0.2% Triton X-100. The sections were then blocked with 5% normal goat serum (S-1000; Vector Laboratories, Newark, CA, USA) for 1 h. After blocking, the sections were incubated overnight at 4 °C with primary antibodies. For double immunofluorescence staining, we used primary antibodies Ki67 and Gli-1 (Supplementary Table S1). On the next day, the sections were washed with TBS containing 0.2% Tween 20 and then incubated with Alexa Fluor 488–conjugated goat anti-mouse immunoglobulin G (IgG) and Alexa Fluor 594–conjugated goat anti-rabbit IgG (Life Technologies) for 1 h at room temperature. Autofluorescence was quenched using an autofluorescence quenching kit (SP-8400-15; Vector Laboratories) for 5 min. Next, the tissue sections were counterstained with 4′,6-diamidino-2-phenylindole (DAPI). Finally, fluorescence signals were detected using a Stellaris 8 confocal microscope (Leica, Wetzlar, Germany).

For fluorescent immunocytochemistry, MPCs were fixed on glass coverslips by incubating them in 4% paraformaldehyde for 10 min at room temperature. Subsequently, the cells were permeabilized with 0.2% Triton X-100–containing TBS for 10 min. Next, the cells were blocked with 5% normal goat serum for 1 h. After blocking, the cells were incubated overnight at 4 °C with primary antibodies (Supplementary Table S1). On the next day, the cells were washed and incubated with Alexa Fluor 488–conjugated goat anti-rabbit IgG or Alexa Fluor 488–conjugated goat anti-mouse IgG (Life Technologies). F-actin was labeled with Alexa Fluor 594 phalloidin (A12381; Invitrogen). Fluorescence signals were detected using a Stellaris 8 confocal microscope (Leica).

### Cytokine array assay

Cytokines present in the human pcMSC secretome were analyzed using antibody array technology (Human Cytokine Arrays C5; RayBiotech, Peachtree Corners, GA, USA). Briefly, the arrays were incubated with the human pcMSC secretome (700 µg/mL) overnight at 4 °C following the manufacturer’s protocol. Signals on the membranes were captured using an ImageQuant LAS 4000 mini system (GE Healthcare, Chicago, IL, USA). Integrated densities were quantified using ImageJ (version 1.53u) (National Institutes of Health, Maryland, USA). The results were normalized by subtracting the surrounding background noise from the signal intensities and comparing the adjusted intensities to the positive control spots.

### Exosomal miRNA extraction, library preparation, and sequencing

Exosome isolation from the human pcMSC secretome, exosomal miRNA extraction, library preparation, and sequencing were performed as described previously [18]. Briefly, pcMSC–CM was centrifuged at 3000 ×*g* for 10 min at 4 °C to remove cell debris. Subsequently, the supernatant was filtered through a 0.22 µm polyethylene terephthalate membrane to eliminate microvesicles. Exosomes were isolated through ultracentrifugation, which was performed using 32-mL thick-wall polycarbonate tubes (355631; Beckman Coulter, Brea, CA, USA). The medium was ultracentrifuged at 100,000 ×*g* for 90 min at 4 °C by using an Optima L-90 K Ultracentrifuge (Beckman Coulter). After the supernatant was carefully discarded, pellets from two tubes were combined and resuspended in ice-cold PBS. A second ultracentrifugation procedure was performed at 100,000 ×*g* for 90 min at 4 °C, and the supernatant was removed. The final exosome pellet was resuspended in 250 µL of ice-cold PBS, yielding crude exosomes.

Total RNA was extracted using TRIzol LS reagent (10296010; Invitrogen) following the manufacturer’s protocol. RNA concentrations were measured at 260 nm by using a NanoDrop spectrophotometer (ND-1000; NanoDrop Technologies, Wilmington, DE, USA). RNA quality was evaluated using a LabChip RNA 6000 kit and a Bioanalyzer 2100 (Agilent Technologies, Santa Clara, CA, USA). Sample libraries were prepared using a QIAseq miRNA Library Kit (Qiagen, Hilden, Germany) following the manufacturer’s instructions and were sequenced using an Illumina instrument and Novaseq 6000 with a 75-cycle single-end read (Illumina, San Diego, CA, USA). In this study, the resultant miRNA sequencing data were accessed in the Gene Expression Omnibus database (accession number: GSE247568) [18].

The sequencing data were demultiplexed using the BCL2FASTQ software (version 2.20; Illumina). High-quality reads were extracted using Trimmomatic, removing reads shorter than 18 nucleotides [27]. Processed reads were analyzed using miRDeep2 [28] and aligned to the GRCh38 reference genome (University of California, Santa Cruz) [29]. To ensure accurate miRNA identification, only reads mapped to the genome in no more than five locations were included for analysis [30]. Normalized miRNA expression was evaluated as reads per million mapped reads, dividing each miRNA’s signal by the total number of mapped reads. TargetScan [31] was used to predict miRNAs targeting genes of interest, focusing on highly expressed candidates with a TargetScan context + + score less than or equal to − 0.1.

### Statistical analysis

All experiments were conducted at least three times. Data were analyzed using GraphPad Prism version 9.3 (GraphPad Software, La Jolla, CA, USA) and are presented in terms of mean ± standard deviation values. An unpaired Student’s *t* test was used for between-group comparisons. One-way analysis of variance with Tukey’s post hoc test was conducted for multigroup comparisons. Statistical significance was set at *P* < 0.05.

## Results

### Characteristics of mouse MPCs

**Fig. 1:**
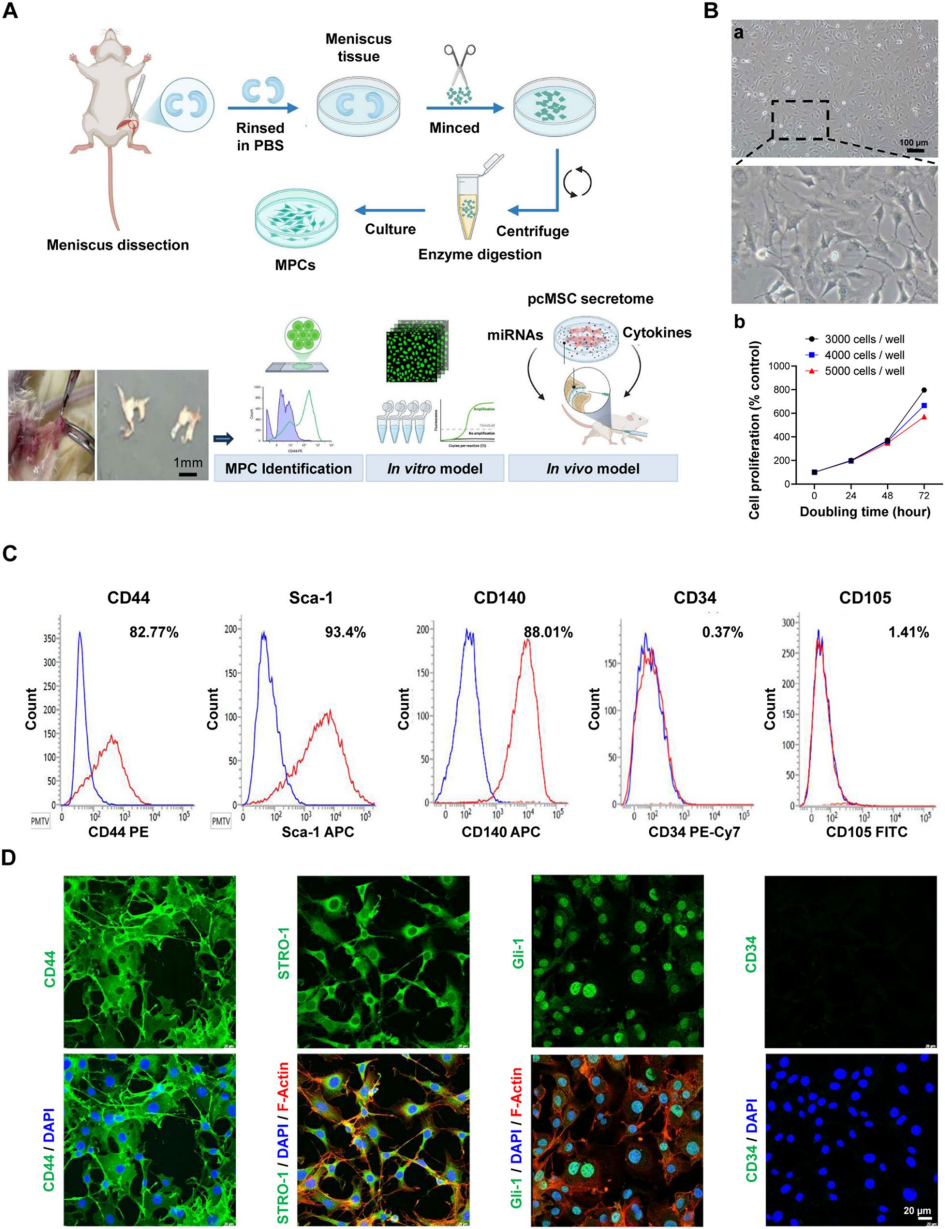
Generation and characterization of mouse MPCs. **A** Culture of mouse MPCs derived from meniscus tissue, followed by MPC identification and experimental analyses in vitro and in vivo models. **B** Cell morphology **a** and doubling time **b** of primary mouse MPCs. Scale bar = 100 μm. **C** Flow cytometry analysis of mouse MPC surface markers: CD44, Sca-1, CD140, CD34, and CD105. **D** Immunofluorescence staining of mouse MPC biomarkers: CD44, Stro-1, and Gli-1 (negative control: CD34). Proteins are shown in green, DAPI is shown in blue, and F-actin is shown in red. Scale bar = 20 μm. Cartoon images were created with BioRender.com. *MPC* meniscus progenitor cell

We successfully established a standard protocol for cultivating mouse MPCs from meniscus tissue (Fig. 1A). These MPCs exhibited a spindle-shaped mesenchymal morphology (Fig. 1Ba), which, along with their proliferation ability (doubling time: 24 h), was maintained for at least 20 passages (Fig. 1Bb).

MPC characteristics were further assessed through flow cytometry and immunocytochemical staining. As shown in Fig. 1C, the MPCs expressed the standard cell surface markers CD44 (82.8%), Sca-1 (93.4%), and CD140 (88.0%) while exhibiting negligible expression of CD34 (< 1%) and CD105 (< 2%). These results were confirmed by immunofluorescence staining, which revealed high expression of the mesenchymal markers CD44 and Stro-1 and the MPC marker Gli-1 but negligible expression of the hematopoietic marker CD34 (Fig. 1D). Taken together, these findings underscore the successful culture of mouse MPCs in vitro.

### Human pcMSC secretome restores viability and promotes self-renewal proliferation in H_2_O_2_-challenged mouse MPCs

**Fig. 2:**
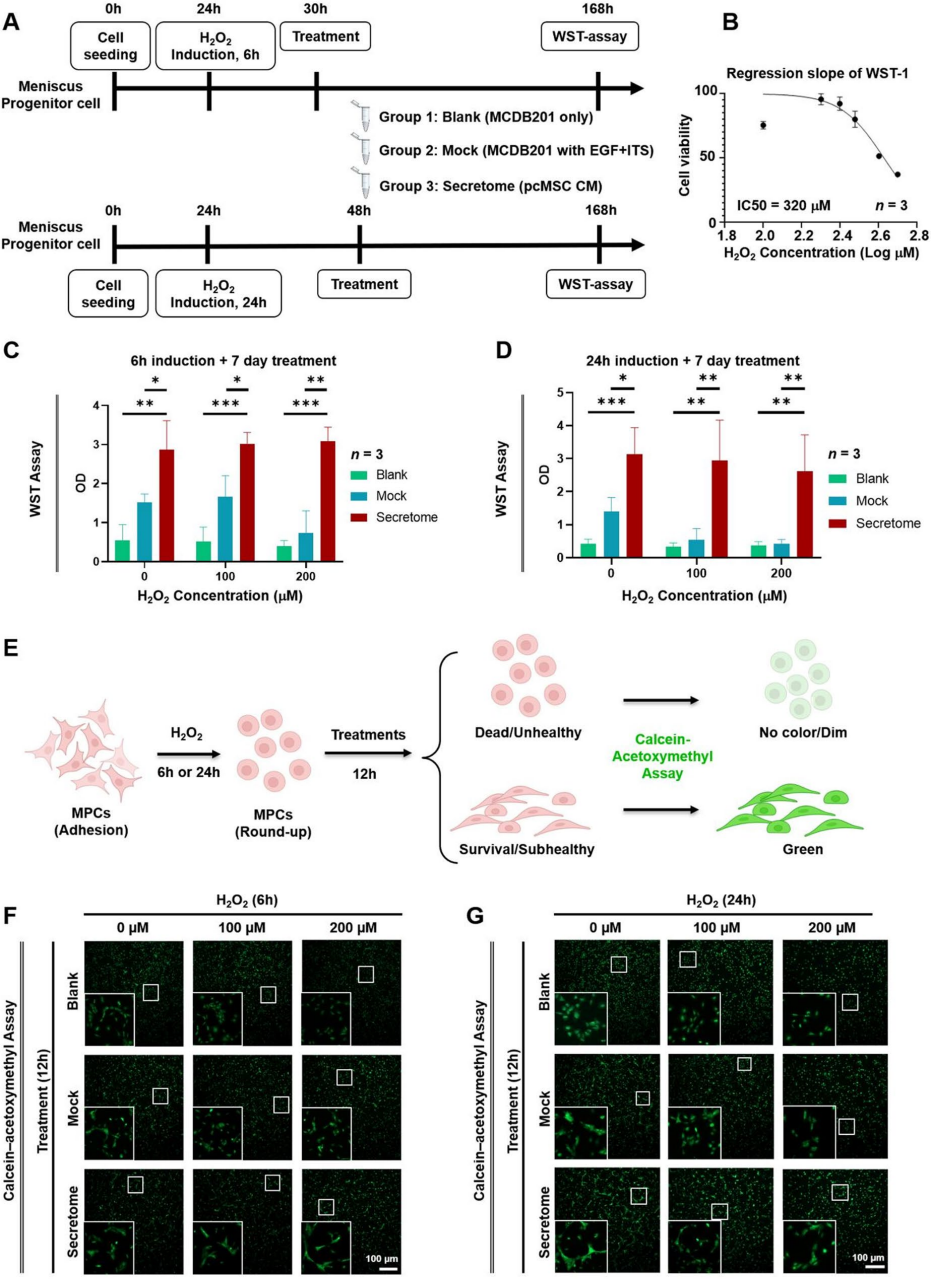
Human pcMSC secretome restores viability and promotes self-renewal proliferation in H_2_O_2_-challenged mouse MPCs. **A** In vitro model of meniscus injury established by challenging mouse MPCs with H_2_O_2_. **B** Half-maximal inhibitory concentration of H_2_O_2_ for mouse MPCs (*n* = 3). **C**, **D** Viability of mouse MPCs subjected to different H_2_O_2_ concentrations (0, 100, and 200 µM) for 6 h (C) or 24 h (D), evaluated through WST assay on day 7. Experimental groups: blank (MCDB201 only, green), mock (MCDB201 with epidermal growth factor (EGF) and insulin–transferrin–selenium solution (ITS), blue), and secretome (pcMSC–CM, red) (*n* = 3). Quantitative analyses are presented. ^*^P < 0.05, ^**^P < 0.01, and ^***^P < 0.001 (one-way analysis of variance with Tukey’s post hoc test). **E** Experimental design of calcein–acetoxymethyl cell viability analysis. **F**, **G** Viability of mouse MPCs subjected to different H_2_O_2_ concentrations (0, 100, and 200 µM) for 6 h (F) or 24 h (G), evaluated through calcein–acetoxymethyl staining after secretome or mock treatment for 12 h. Live cells are shown in green. Cartoon images were created with BioRender.com. *pcMSC* placenta-derived mesenchymal stem cell, *MPC* meniscus progenitor cell

Primary mouse MPCs were used to examine the effect of the human pcMSC secretome on MPC apoptosis. To this end, an in vitro model of MI was established by challenging mouse MPCs with H_2_O_2_; the experimental design is presented in Fig. 2A. The efficacy of the human pcMSC secretome in restoring the viability and proliferative ability of the H_2_O_2_-challenged MPCs was evaluated through a WST assay. All the following experiments were verified in three independent experiments. First, the half-maximal inhibitory concentration (IC50) of H_2_O_2_ was 320 µM (Fig. 2B). As mentioned earlier, the H_2_O_2_-challenged MPCs were divided into three groups on the basis of the culture media: blank, mock, and secretome groups.

Cell viability was analyzed through a WST assay, and revealed that the MPCs viability of the secretome group was significantly higher than that of in blank and mock groups with short-term (6 h) or long-term (24 h) H_2_O_2_-challenges (Fig. 2C and D). Our calcein–acetoxymethyl cell viability assay (Fig. 2E) also revealed that the degree of viability (in green) was higher in the secretome group than in the blank and mock groups (Fig. 2F and G). Moreover, compared to blank and mock groups, MPCs of the secretome group still showed an adherent morphology in 100 µM and 200 µM of H_2_O_2_ challenges, indicating that the pcMSC secretome could diminish cell injury and restore cell health (Fig. 2F and G, inset boxes).

**Fig. 3:**
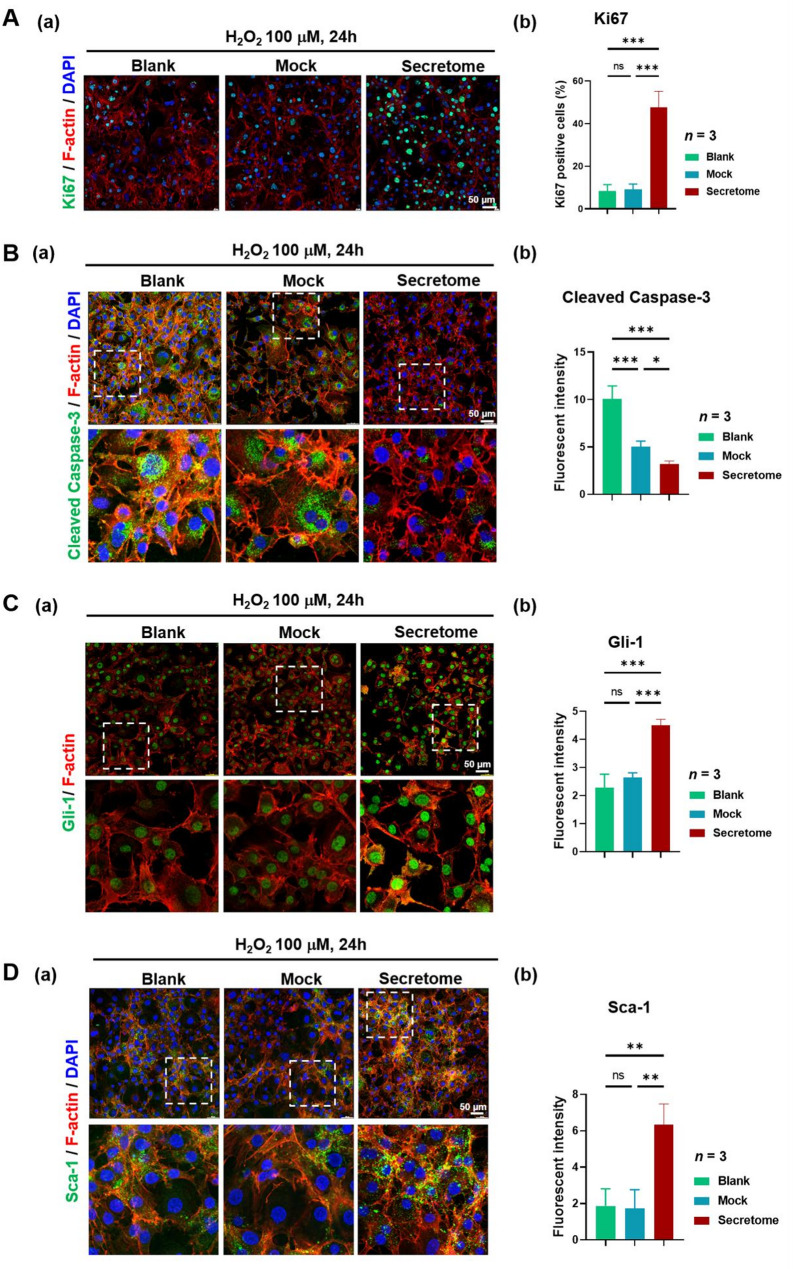
Human pcMSC secretome mitigates H_2_O_2_-induced MI in mouse MPCs. Mouse MPCs were exposed to 100 µM H_2_O_2_ for 24 h. Experimental groups: blank (MCDB201 only, green column), mock (MCDB201 with epidermal growth factor (EGF) and insulin–transferrin–selenium solution (ITS), blue column), and secretome (pcMSC–CM, red column). The expression levels of **A** Ki67, **B** cleaved Caspase-3, **C** Gli-1, and **D** Sca-1 proteins were determined through immunofluorescence staining and quantitative analysis (*n* = 3). Ki67, cleaved Caspase-3, Gli-1, and Sca-1 are shown in green; DAPI is shown in blue; and F-actin is shown in red. Scale bar = 50 μm. ^*^P < 0.05, ^**^P < 0.01, and ^***^P < 0.001 (one-way analysis of variance with Tukey’s post hoc test). *ns* no significance

The ability of the human pcMSC secretome to restore proliferation and suppress apoptosis in H_2_O_2_-treated MPCs was evaluated through immunofluorescence staining for Ki67 (cell proliferation marker; Fig. 3A) and cleaved Caspase-3 (apoptosis marker; Fig. 3B). Notably, the secretome significantly activated the self-renewal proliferation of MPCs, as indicated by coimmunostaining for Gli-1 (MPC marker; Fig. 3C), Sca-1 (MPC marker; Fig. 3D) and Ki67 (Fig. 3A). The results of our quantitative and statistical analyses are presented in the right panel of each figure (Fig. 3). Collectively, these findings suggest that the human pcMSC secretome effectively restores cell viability and promotes self-renewal proliferation in H_2_O_2_-challenged MPCs.

### Human pcMSC secretome ameliorates DMM-induced MI in mice

We previously demonstrated that the pcMSC secretome restores premature ovarian insufficiency and circadian rhythm disruption induced by the chemotherapeutic agent cyclophosphamide [18]. In the present study, we extend the application of the pcMSC secretome to the treatment of DMM-induced MI.

**Fig. 4:**
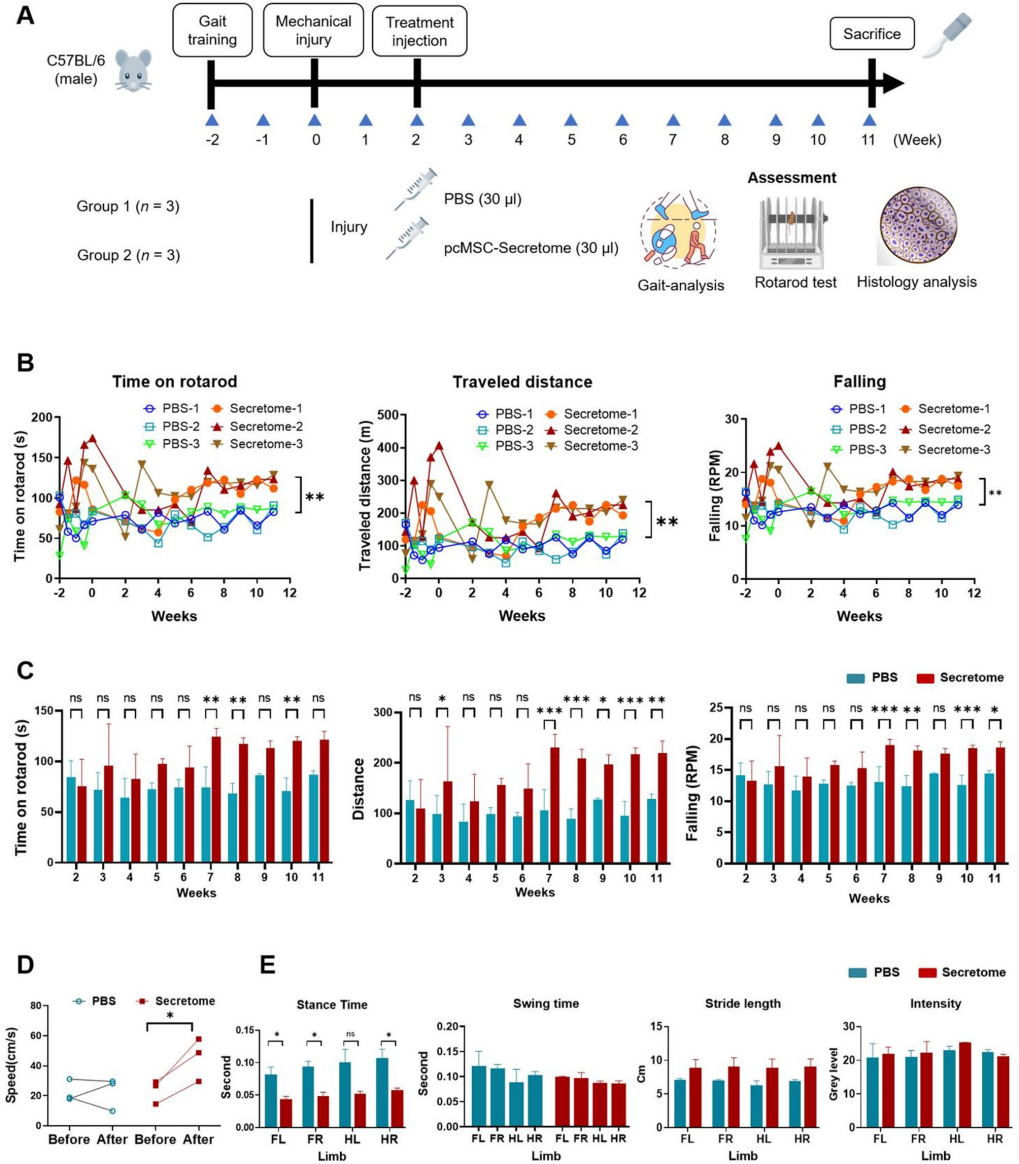
Human pcMSC secretome mitigates DMM-induced MI in mice. **A** Mouse model of DMM-induced MI. **B**, **C** Functional activity in the PBS (*n* = 3, blue–green color) and secretome (*n* = 3, orange–golden color) groups, assessed in terms of time spent on the rotarod, traveled distance, and falling revolutions per minute (RPM); the tests were conducted every week from weeks − 2 to 11. **D** Walking speed analyzed using GaitLab. **E** Stance time, swing time, stride length, and intensity analyzed using GaitLab. ^*^P < 0.05, ^**^P < 0.01, and ^***^P < 0.001 (one-way analysis of variance with Tukey’s post hoc test). Cartoon images were created with BioRender.com. *pcMSC* placenta-derived mesenchymal stem cell, *PBS* phosphate-buffered saline, *FL* forelimb left, *FR* forelimb right, *HL* hindlimb left, *HR* hindlimb right, *ns* no significance

The MI mice were divided into two groups that received local injections of either PBS (PBS group) or secretome (Secretome group). The experimental design is presented in Fig. 4A. Functional evaluations were conducted through gait and rotarod tests, followed by histological and immunohistochemical analyses (Fig. 4A).

Briefly, the effect of the pcMSC secretome on mice was evaluated using a rotarod test, which measured the time spent on a rod. The results indicated that DMM surgery significantly reduced test performance parameters, such as time spent on the rotarod, travel distance, and falling revolutions per minute (RPM) (Fig. 4B). This impaired performance persisted until the pcMSC secretome was injected. Starting from week 7, the secretome effectively mitigated DMM-induced deficits, leading to gradual improvements in rotarod performance, travel distance, and falling RPM until week 11 (Fig. 4B). Significant between group differences in weekly performance are presented in Fig. 4C and Supplementary Fig. S1 (red column vs. blue column).

The therapeutic effects of the pcMSC secretome were evaluated through a spatiotemporal gait analysis. The results indicated that the secretome significantly improved walking speed and stance time (Fig. 4D and E); additionally, a trend toward recovery was evident from reductions in swing time and stride length. Compared with the PBS group, the secretome group exhibited a similar level of intensity as well as reduced swing time and increased stride length indicated that although leg movement was unimpaired, the injured knee joint caused difficulty in each step (Fig. 4E). The above experiments were verified in three independent experiments.

### Human pcMSC secretome repairs MI in mice by activating the proliferation and suppressing the apoptosis of endogenous MPCs

**Fig. 5:**
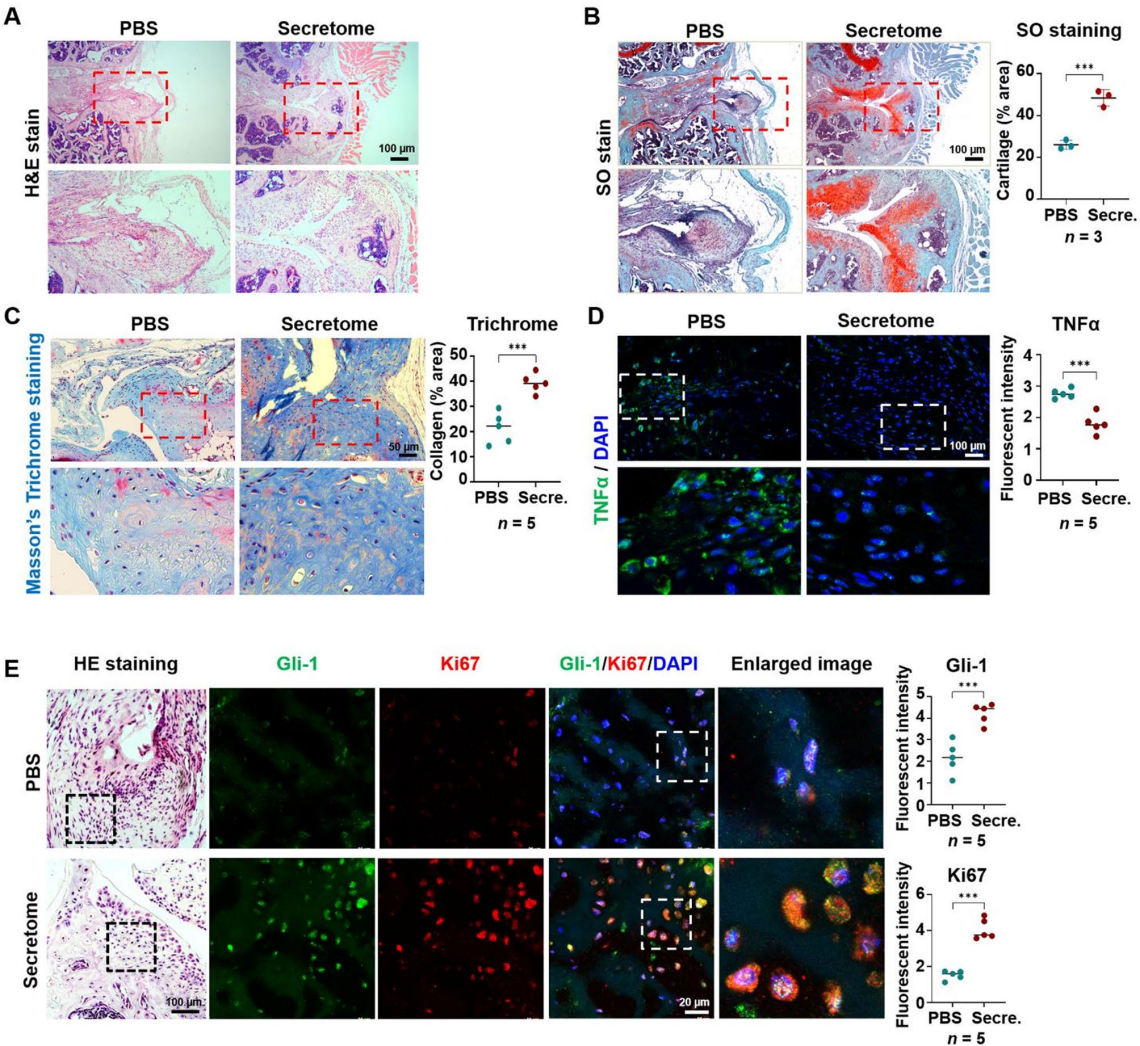
Human pcMSC secretome repairs DMM-induced MI by alleviating inflammation and activating endogenous MPC proliferation. Efficacy of the pcMSC secretome in repairing DMM-induced MI in mice. The results of **A** hematoxylin and eosin (H&E) staining, **B** safranin-O (SO) staining (*n* = 3), and **C** Masson’s trichrome (*n* = 5) staining are presented. Confocal microscopic images of **D** immunofluorescence staining (*n* = 5) of the inflammation marker tumor necrosis factor-α (green) and **E** coimmunostaining of the endogenous MPC marker Gli-1 (green) and the proliferation marker Ki67 (red). DAPI is shown in blue (*n* = 5)

To further investigate the therapeutic benefits of the pcMSC secretome, histological analysis and immunohistochemical staining were performed, enabling the evaluation of meniscus repair, immunomodulation, and endogenous MPC proliferation. H&E staining revealed that the secretome group exhibited intact tissue with a smooth surface, whereas the PBS group exhibited fragile tissue with an uneven structure (Fig. 5A). Compared with the PBS group, the secretome group exhibited a higher abundance of chondrocytes and ECM, as indicated by SO staining (Fig. 5B, *P* < 0.001). Both H&E and SO staining revealed that the secretome promoted chondrocyte and ECM proliferation, leading to a highly organized hierarchical structure resembling a healthy meniscus. This intact structure likely contributed to the superior functional performance of the secretome group in both the rotarod and gait tests by improving structural integrity and load-bearing capacity. Furthermore, Masson’s trichrome staining, which assesses collagen fiber distribution, suggested that the pcMSC secretome expanded the positive staining area and mitigated articular cartilage surface degradation. In addition, the intense staining of the meniscus in the secretome group indicated an increased concentration of collagen fibers, highlighting a more integrated and robust meniscus structure in the secretome group than in the PBS group (Fig. 5C, *P* < 0.001).

Immunohistochemical staining was performed to evaluate the effects of the pcMSC secretome on immunomodulation and endogenous MPC proliferation in mice with DMM-induced MI. As shown in Fig. 5D, the pcMSC secretome significantly reduced the levels of tumor necrosis factor-α (TNF-α) in mice with DMM-induced MI. This finding supports the role of the human pcMSC secretome in immunomodulation and meniscus repair (Fig. 5D, *P* < 0.001).

Ki67/Gli-1 coimmunostaining indicated the efficacy of the pcMSC secretome in activating endogenous MPC proliferation. Secretome treatment markedly increased the expression of both Gli-1 and Ki67 proteins, which presented in the meniscus tissue of mice with DMM-induced MI (*P* < 0.001) (Fig. 5E, Supplementary Fig. S2). Given that Gli-1 is a well-documented transcription factor that regulates MPCs [16], our findings strongly suggest that the pcMSC secretome promotes the proliferation of MPCs and the regeneration of DMM-damaged meniscal tissue in vivo.

### Growth factors and exosomal miRNAs in the human pcMSC secretome May mediate its therapeutic effects against DMM-induced MI

To elucidate the potential mechanisms underlying the effects of the pcMSC secretome, we conducted a cytokine array assay and an exosomal miRNA analysis to identify key paracrine factors and exosomal components.

**Fig. 6:**
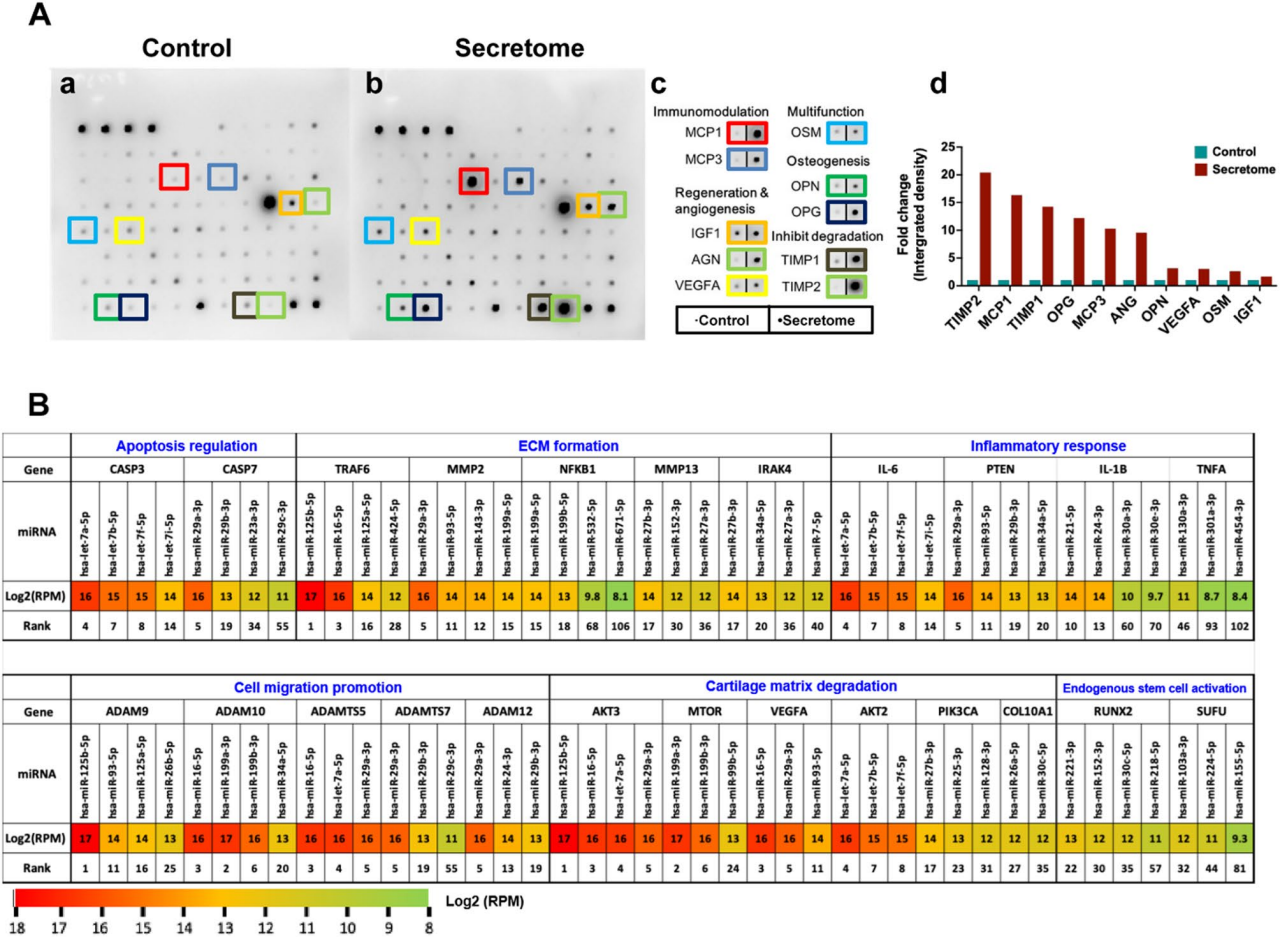
Key factors present within the pcMSC secretome. **A** A cytokine array assay was performed to analyze the protein components of the secretome derived from a nonconcentrated medium pool (passage-8 to passage-11 pcMSCs; total protein concentration: 700 µg/mL). **a** PBS (control, MCDB201 only). **b** Secretome (pcMSC–CM). **c** Top ten abundant cytokines in the PBS and secretome groups. **d** Quantitative data corresponding to the findings presented in **c**. **B** Heatmap predicting miRNA–gene interactions. TargetScanHuman (version 8.0) was used to calculate targetability scores by selecting miRNAs with a TargetScore context + + score of less than or equal to − 0.1. Genes of interest were categorized as follows: apoptosis regulation, ECM formation, inflammatory responses, cell migration promotion, cartilage matrix degradation, and endogenous stem cell activation. The expression of miRNAs is presented as log2 RPM, with the RPM ratio columns indicating changes in miRNA expression. *ECM* Extracellular matrix, *RPM* reads per million

As shown in Fig. 6A, the cytokine array assay revealed several cytokines in the pcMSC secretome that likely contributed to inhibition of ECM/cartilage matrix degradation (TIMP1 and TIMP2), immunomodulation (MCP1 and MCP3), osteogenesis (OPN and OPG), and angiogenesis and regeneration (IGF-1, ANG, and VEGFA). A basal medium without pcMSCs was used as a negative control (control group) (Fig. 6Aa-Ac). As shown in Fig. 6Ad, the expression levels of various cytokines were higher in the secretome group than in the control group. These findings highlight the key roles of these cytokines in immunomodulation, osteogenesis, regeneration, angiogenesis, and ECM/cartilage matrix preservation.

We previously published the miRNA profiles of pcMSC-derived exosomes [18]. In the present study, we investigated key miRNA candidates involved in meniscus repair (Fig. 6B). These miRNA candidates regulate the expression of genes associated with apoptosis (caspase-3 [*CASP3*] and caspase-7 [*CASP7*]), ECM formation (TNF receptor–associated factor 6 [*TRAF6*] and *MMPs*), anti-inflammatory responses (*PTEN* and interleukin-6 [*IL-6*]), cell migration (*ADAMTS*s and *ADAMs*), anti-cartilage matrix degradation (*mTOR*, *COL10A1*, and *AKT*s), and endogenous stem cell activation (*SUFU* and *RUNX2*).

In this study, miRNA profiling revealed significant associations between the miRNA candidates and multiple functions. Notably, some miRNAs regulated two or more target genes. For example, hsa-miR-125b-5p and hsa-miR-16-5p were involved in ECM formation, anti-cartilage matrix degradation, and cell migration; hsa-let-7a-5p and hsa-miR-29a-3p were associated with anti-cartilage matrix degradation, cell migration, apoptosis prevention, and anti-inflammatory responses; hsa-miR-199a-3p contributed to anti-cartilage matrix degradation and cell migration; hsa-let-7b-5p was associated with *AKT2*, *AKT3*, *ADAMTS5*, *CASP3*, and *IL-6*; and hsa-miR-199b-3p regulated *mTOR* and *ADAM10*. As shown in Fig. S3, we identified several potential exosomal miRNA candidates, including miR-29a-3p, miR-27b-3p, miR-93-5p, miR-16-5p, and let-7a-5p, which targeting specific gene expressions to significantly decrease the injury response of mouse MPCs (Supplementary Fig. S3 and Table S2). Taken together, these findings suggest that the exosomal miRNAs present within the pcMSC secretome playing multifaceted roles in meniscus repair via anti-apoptosis, anti-inflammation, anti-cartilage matrix degradation, and promoting ECM formation, all highlighting its potential for MI therapy.

## Discussion

**Fig. 7:**
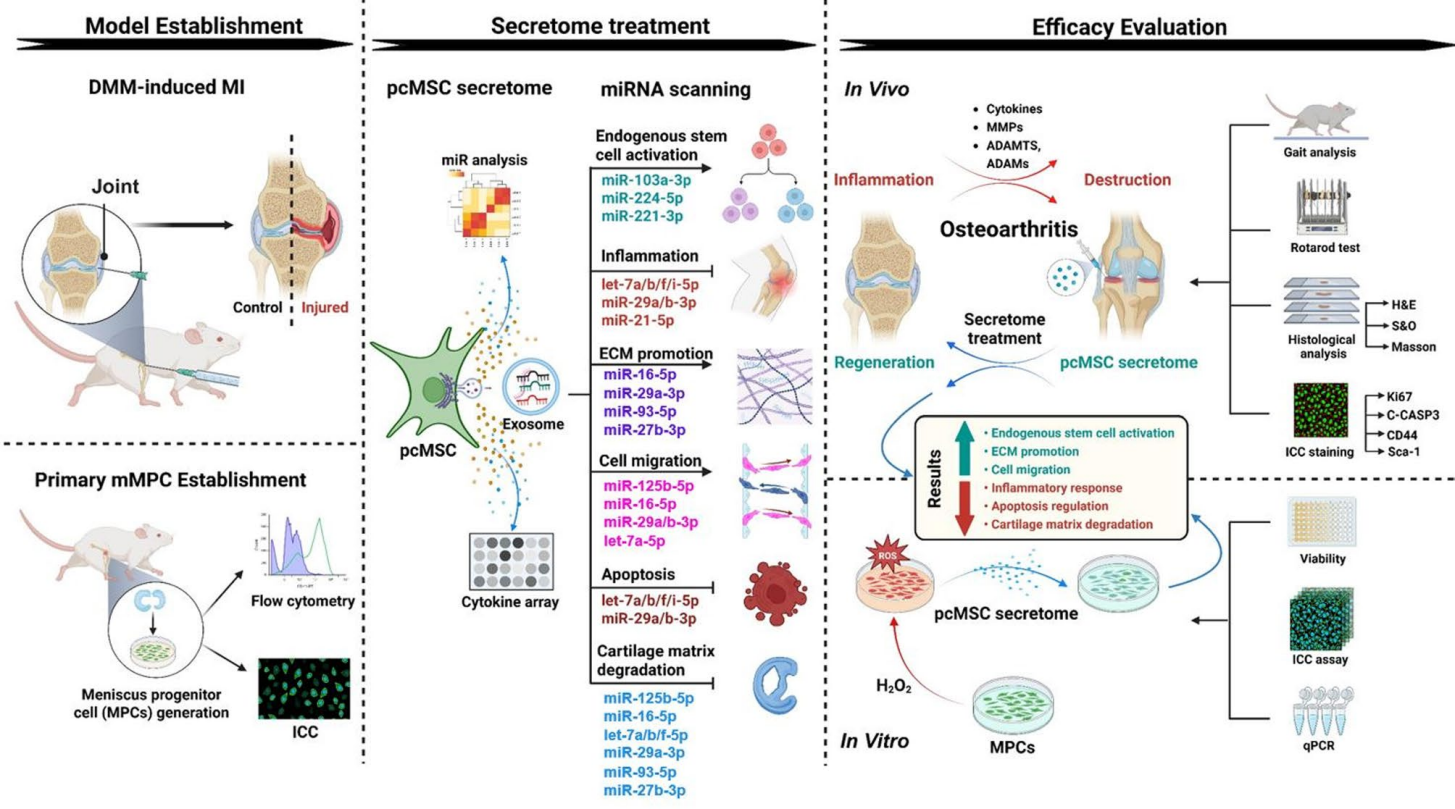
Mechanisms underlying the therapeutic effects of the human pcMSC secretome in vitro and in vivo. (Left) Model establishment: An in vivo model of MI was established through DMM surgery, and an in vitro model of MI was established through H_2_O_2_-challenged primary MPCs. (Middle) Key factors mediating the therapeutic effects of the human pcMSC secretome: cytokines, growth factors, and exosomal microRNAs. (Right) Potential therapeutic mechanisms underlying the repair of H_2_O_2_-induced MI and DMM-induced MI by the pcMSC secretome treatment. Cartoon images were created with BioRender.com. *pcMSC* placenta-derived mesenchymal stem cell, *MI* meniscus injury, *MPC* meniscus progenitor cell, *mMPC* mouse MPC, *DMM* destabilization of the medial meniscus

To the best of our knowledge, this is the first study to demonstrate the therapeutic potential of the human pcMSC secretome for treating DMM-induced MI. We used both in vivo and in vitro models of MI to study the effects of the secretome on endogenous MPCs. Our results suggest that these effects are mediated through the paracrine factors and exosomal miRNAs present within the pcMSC secretome. These findings may provide valuable insights for researchers developing stem cell-derived secretome cell-free therapies for individuals with exercise-induced MI (Fig. 7).

MI is a common problem, particularly among athletes and older adults. The limitations and suboptimal outcomes of current treatments have been recognized for years. A key intrinsic limitation of meniscus repair is the poor vascularization of the capsular and synovial tissues [32]. The peripheral region—known as the red–red zone—has a rich vascular supply, whereas the inner regions—known as the red–white and white–white zones—are relatively avascular; the lack of blood supply restricts the availability of proliferative cytokines and growth factors [33].

Traditional therapeutic strategies for MI suffer major challenges. Meniscectomy has been a quick and widely used to alleviate pain and improve mobility in patients with MI [34] or osteoarthritis [35]. However, given that the biomechanical consequences of total meniscectomy have become better understood, new strategies aimed at preserving as much of the meniscus as possible have gained traction. Thus, surgical approaches such as partial meniscectomy, meniscus repair, and meniscus transplantation have been increasingly adopted.

Although partial meniscectomy is associated with improved outcomes [36], long-term studies have indicated that the degeneration of the remaining tissue often leads to osteoarthritis [37]. Consequently, surgeons have been exhibiting a preference toward meniscus repair surgery, which yields long-term outcomes that surpass those of total or partial meniscectomy [38]. Sutures and screws are commonly used in meniscus repair, a procedure whose success strongly depends on the vascular supply at the repair site. Healing is more likely in vascular zones, whereas the outcomes in avascular zones remain less satisfactory [39]. Meniscus transplantation is another option for meniscus repair; however, this approach poses challenges related to allograft availability. Moreover, the lack of well-designed randomized controlled trials limits evidence supporting the effectiveness of meniscus transplantation [40].

To overcome the aforementioned therapeutic limitations, recent preclinical studies have proposed cell-based regenerative therapy [41]. BMMSCs exhibit greater efficacy than that of platelet-rich plasma in treating avascular zone injury after suturing, highlighting the therapeutic potential of MSCs in poorly vascularized regions [42]. Additionally, human umbilical cord-derived MSCs (ucMSCs) have been reported to improve sarcopenia-related skeletal muscle atrophy and dysfunction through anti-apoptosis, anti-inflammatory, and mitochondrial biogenesis mechanisms [43]. Similarly, three-dimensional adipose-derived stem cells effectively promote meniscus healing in rabbit models, but the precise mechanisms remain unclear [44]. Histological evidence suggests that SMSCs adhere to injury sites and facilitate meniscus repair [45–47]. Chondroprogenitor cells respond to SDF-1 and promote collagen bridging across inner meniscus tears; consequently, the SDF-1/CXCR4 signaling pathway plays a crucial role in the migration of meniscus cells [48].

Multiple clinical trials evaluate the safety and efficacy of MSCs in meniscus repair, for instance, Table 1 lists ongoing clinical trials exploring the therapeutic benefits of MSCs (Table 1). A single-center, prospective, open-label clinical trial indicated that BMMSCs combined with a scaffold exhibited improved efficacy in treating torn avascular MI [49]. Another trial reported enhanced outcomes when surgical repair was combined with SMSC transplantation [50]. These findings suggest that cell therapy holds clinical value and can promote meniscus regeneration in humans.

**Table 1:** Current clinical trials evaluating meniscus injury therapy with mesenchymal stem cells and their secretome/exosomes

NCT number	Phase	Trial name	Time of completion
NCT02033525	I/II	Mesenchymal Stromal Cells for Degenerative Meniscus Injury	2018
NCT03955497	I/II	Effectiveness of Autologous Adipose-Derived Stem Cells in the Treatment of Knee Cartilage Injury	2022
NCT05081921	I/II	Clinical Trial to Evaluate Safety and Efficacy of MesoCellA-Ortho Tissue-Engineered Advanced Therapy Product in Patients with Osteoarthrosis and Civilisation Diseases (BioMiStem-CT)	2024
NCT05261360	II	Clinical Efficacy of Exosome in Degenerative Meniscal Injury	2025

*NCT* national clinical trial

Notably, previous clinical trials have presented several limitations, such as small sample sizes, short follow-up periods, and signal-arm designs (no control group). Only Olivos-Meza et al. [51] included a control group in their trial but found no benefit from autologous CD90^+^ MSC treatment. Both autologous and allogeneic methods have limitations. For instance, the autologous method primarily relies on patient viability and surgical technique, introducing variability in outcomes and complicating conclusions [52]. In addition, the allogeneic method is associated with concerns regarding safety and immune rejection [21, 53]. Thus, large multicenter clinical studies and meta-analyses are urgently required to confirm the efficacy of cell therapy for MI.

In addition to MSCs, the MSC-derived secretome is effective in the treatment of MI, offering advantages such as effective penetration into narrow joint spaces and sustained retention within the meniscus [34, 54]. Moreover, the secretome exhibits strong immunomodulatory properties, addressing concerns regarding immune rejection [55]. Therefore, the MSC-derived paracrine factors and extracellular vesicles, including exosomes, which facilitate the modulation of TNF-α-induced pro-inflammatory [56], promote the production of proteoglycans and collagen II [57], and ameliorate muscle atrophy via mitochondrial biogenesis, anti-apoptosis, and protein anabolism mechanisms [58]. In the context of MI, MSC-derived exosomes mitigate the risk of osteoarthritis by enhancing chondrocyte proliferation, promoting migration, and inhibiting apoptosis [59, 60]. These exosomes also prevent cartilage degradation [59], protect cells against oxidative stress [61], and modulate signaling cascades such as the PI3K/AKT and MAPK/ERK pathways [62]. However, to date, few studies have investigated the efficacy of the MSC secretome and MSC-derived exosomes in regulating endogenous MPCs. Moreover, the key bioactive factors present within the MSC secretome remain unclear. This study represents a major advancement in research into stem cell therapy for MI because it involves a comprehensive investigation into the therapeutic potential of the pcMSC secretome in meniscus repair. Although previous studies have explored various aspects of MSC therapy, this study is unique because it combines in vivo and in vitro models to elucidate the mechanisms through which the pcMSC secretome activates the proliferation and suppresses the apoptosis of endogenous MPCs.

Our in vivo model revealed the marked efficacy of the pcMSC secretome in meniscus repair; this finding was corroborated by our rotarod and gait test results (Fig. 4). The key processes involved in meniscus repair and tissue regeneration included the modulation of niche immune environment, the activation of endogenous MPC proliferation, and the suppression of apoptosis. Our in vitro model revealed that the pcMSC secretome significantly inhibited H_2_O_2_-induced apoptosis (Fig. 3B) and promoted MPC proliferation (Fig. 3A and C, and D), as indicated by the proliferation marker Ki67 and the MPC markers Gli-1 and Sca-1. Supporting these findings, H&E and SO staining highlighted marked improvements in tissue morphology, as evident from enhanced proteoglycan content (Fig. 5B). Masson’s trichrome staining revealed increased collagen deposition (Fig. 5C). Immunohistochemical staining revealed weak signals for TNF-α (Fig. 5D) and strong positive signals for Gli-1 and Ki67 (Fig. 5E) in MI tissue following secretome treatment. These results implicate the pcMSC secretome in the modulation of niche immune environment and the activation of endogenous MPC proliferation.

The MSC secretome comprises elements such as extracellular vesicles, miRNAs, cytokines, and proteins. These elements play crucial roles in tissue healing, particularly in meniscus repair. The secretome promotes meniscus healing by enhancing the production of key factors such as IL-6, MMP-3, MMP-13, and VEGF [63, 64]. It also regulates cellular processes such as proliferation, apoptosis, and ECM degradation through various signaling cascades [65].

Cytokine array assays and miRNA profiling revealed several candidates that mediate the function of the human pcMSC secretome in meniscus repair. These candidates involving in multiple aspects especially for the regulate endogenous stem cell activation (hsa-miR-221-3p and hsa-miR-152-3p), anti-inflammatory responses (MCP1 and MCP3), ECM formation (hsa-miR-125b-5p), apoptosis (hsa-let-7a-5p and hsa-let-7b-5p), regeneration and angiogenesis (IGF-1, ANG, and VEGFA), and inhibit ECM degradation (TIMP1 and TIMP2). Some miRNAs play multifaceted roles. For instance, hsa-let-7a-5p and hsa-miR-29a-3p, which are abundant in the pcMSC secretome, prevent cartilage matrix degradation, promote cell migration, suppress apoptosis, and alleviate inflammation. Similarly, hsa-miR-125b-5p and hsa-miR-16-5p, which are highly expressed in the secretome, prevent cartilage matrix degradation and promote cell migration and ECM formation. Furthermore, hsa-miR-199a-3p prevents cartilage matrix degradation and promotes cell migration. Notably, hsa-let-7b-5p regulates *AKT2*, *CASP3*, and *IL-6*, whereas hsa-miR-199b-3p regulates *mTOR* and *ADAM10*.

Recently, miRNAs have emerged as key markers of meniscal degeneration, osteoarthritis progression, and meniscus healing [66]. Several miRNAs, such as miR-140, miR-27b, miR-16, miR-22, and miR-146a, serve as genetic markers of osteoarthritis. Moreover, miR-29a-3p, let-7a-5p, miR-27b-3p, miR-16, and others have been validated to play therapeutic roles in soft tissue injuries in sports medicine [63]. Notably, miRNAs in the synovial fluid of patients with osteoarthritis originate not only from cartilage but also from the synovium and menisci, which highlights their potential as biomarkers for monitoring osteoarthritis progression [67, 68]. Long et al. [68] explored the molecular aspects of the meniscus and suggested that miRNAs can serve as biomarkers of osteoarthritis development. Some miRNAs induce gene expression. For instance, miR-210 accelerates meniscus healing by upregulating the expression of *COL2A1*, *VEGF*, and *FGF2* [69]. The level of miRNA expression in the meniscus varies in a concentration- and time-dependent manner when induced by inflammatory cytokines; therefore, miRNA profiling may facilitate the early detection of osteoarthritis [67]. Li et al. [70] reported that miR-490-5p attenuated the chondrogenesis of human adipose-derived stem cells and accelerated the degradation of cartilage by activating the PI3K/AKT pathway by targeting the PITPNM1.

To further examine the effects of various miRNAs, we conducted a miRNA analysis to explore potential mechanisms at the level of gene expression. We identified specific miRNAs in the pcMSC secretome that regulate the following processes: apoptosis (*CASP3* and *CASP7*), ECM formation (*TRAF6* and *MMP2*), inflammatory responses (*IL-6*, *PTEN*, *IL-1β*, and *TNF-α*), cell migration (*ADAM* family), cartilage matrix degradation (*AKT2*, *AKT3*, *mTOR*, and *VEGFA*), and endogenous stem cell activation (*SUFU* and *RUNX2*). Our analysis highlighted key miRNAs involved in activating endogenous MPC proliferation, suppressing apoptosis, and promoting cell migration and ECM formation. The inhibition of inflammation, chondrocyte degradation, and apoptosis by these miRNAs likely led to the favorable outcomes observed in our in vivo study. Therefore, the pcMSC secretome appears to be a promising candidate for meniscus repair and osteoarthritis treatment.

Studies on meniscus repair with MSCs have primarily focused on the proliferative effects of direct MSC injections on chondrocytes and meniscus tissue. Treatment efficacy has often been evaluated solely through histological analysis or meniscus morphology. Horie et al. [71, 72] and Caminal et al. [71, 72] conducted animal studies using intra-articular MSC injections and demonstrated the efficacy of this approach in meniscus regeneration and cartilage protection. Injecting adipose-derived MSCs into meniscus allograft tissue activates cell proliferation, promotes cell migration, and improves cell survival within allografts [73]. In addition, injecting allogeneic human BMMSCs enhances the anti-inflammatory and regenerative capacities of the meniscus and cartilage, leading to the inhibition of fibrotic and hypertrophic processes. However, few studies have explored the role of the MSC secretome or exosomal miRNAs in regulating the activation of endogenous MPCs. The absence of in vitro MPC models and in vivo biomechanical preclinical data precludes the assessment of whether the healing meniscus meets the mechanical demands required for satisfactory motor function [72, 74].

To address the aforementioned limitations, we developed primary mouse MPCs into an in vitro model of MI. In addition, we established an in vivo model of DMM-induced MI. This dual approach offered a robust framework for understanding the regenerative capabilities of the pcMSC secretome. Even though this study fills the gaps in the literature by clarifying the role of the pcMSC secretome in the regulation of endogenous MPCs, this study has several limitations, and we will aim to uncover more precise molecular regulatory mechanisms by gain-of-function and loss-of-function experiments targeting specific cytokines or miRNAs in a mouse MI model in the future.

## Conclusion

To the best of our knowledge, this is the first study using both in vitro model of H_2_O_2_-challenged MPCs and in vivo model of mechanically DMM-induced MI to demonstrate the therapeutic efficacy of the human pcMSC secretome through activating the meniscus progenitor cells in meniscus regeneration. Among the potentially essential factors present within the secretome are growth factors and exosomal miRNAs; these factors regulate the activation of endogenous MPCs (by promoting MPC proliferation and suppressing apoptosis), the immunomodulation of injured tissue (by alleviating inflammation), and the reconstruction of the ECM (by preventing cartilage matrix degradation and promoting cell migration). The exosomal miRNA (miR-29a-3p, miR-27b-3p, miR-93-5p, miR-16-5p, and let-7a-5p) effectively modulated ECM formation, anti-apoptosis, anti-inflammation, and anti-cartilage matrix degradation to mitigate meniscus cell injury. Overall, our findings may guide the development of effective stem cell therapies for patients with exercise-induced meniscus injuries.

## Supplementary Information

Below is the link to the electronic supplementary material.

Supplementary Material 1.

## Data Availability

The miRNA sequencing data were accessed in the Gene Expression Omnibus (GEO) repository (accession number: GSE247568) [18]. All data generated or analyzed during this study are included in this published article and its supplementary information files.

## Abbreviations

ADMSCs: Adipose-derived mesenchymal stem cells
BMMSC: Bone marrow–derived mesenchymal stem cell
CM: Conditioned medium
DAPI: 4′,6-Diamidino-2-phenylindole
DMM: Destabilization of the medial meniscus
ECM: Extracellular matrix
H&E: Hematoxylin and eosin
IL: Interleukin
MI: Meniscus injury
miRNA: MicroRNA
MMP: Matrix metallopeptidase
MPC: Meniscus progenitor cell
MSC: Mesenchymal stem cell
PBS: Phosphate-buffered saline
pcMSC: Placenta-derived mesenchymal stem cell
SMSC: Synovial mesenchymal stem cell
SO: Safranin-O
TBS: Tris-buffered saline
TNF-α: Tumor necrosis factor-α
TRAF6: TNF receptor associated factor 6
ucMSC: Umbilical cord-derived mesenchymal stem cell
