# Supplementary material for: Secretome from human placenta-derived mesenchymal stem cells repairs mechanically induced meniscus injury in mice by activating the proliferation and suppressing the apoptosis of endogenous meniscus progenitor cells
Source: Stem Cell Res Ther. 2025 Oct 14;16:565. doi: 10.1186/s13287-025-04688-6 (PMC12523042; doi:10.1186/s13287-025-04688-6)
Supplement: Supplementary file 1 — Supplementary Material 1. [file 13287_2025_4688_MOESM1_ESM.docx]

**Supplementary information**


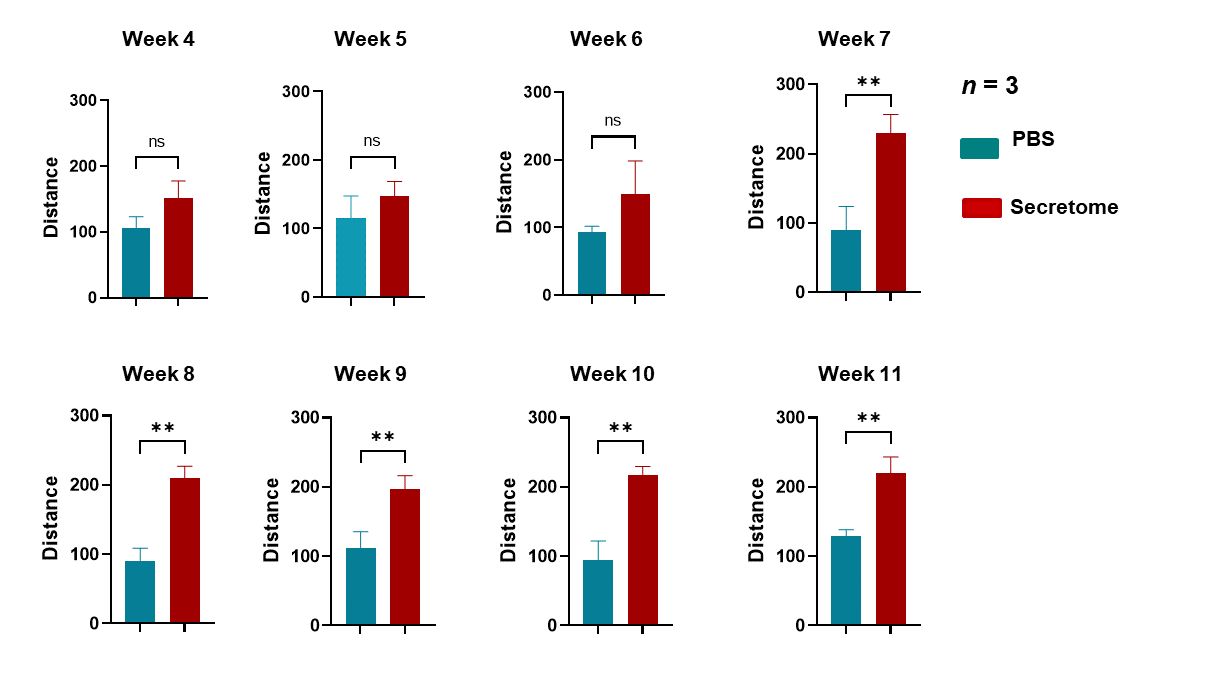


**Supplementary Figure S1. Weekly rotarod test results after week 4. (Related to main Figure 4.)**

Functional activity in the PBS (*n* = 3) and secretome (*n* = 3) groups, assessed in terms of traveled distance. ^*^*P* < 0.05 and ^**^*P* < 0.01 (Student’s *t* test).


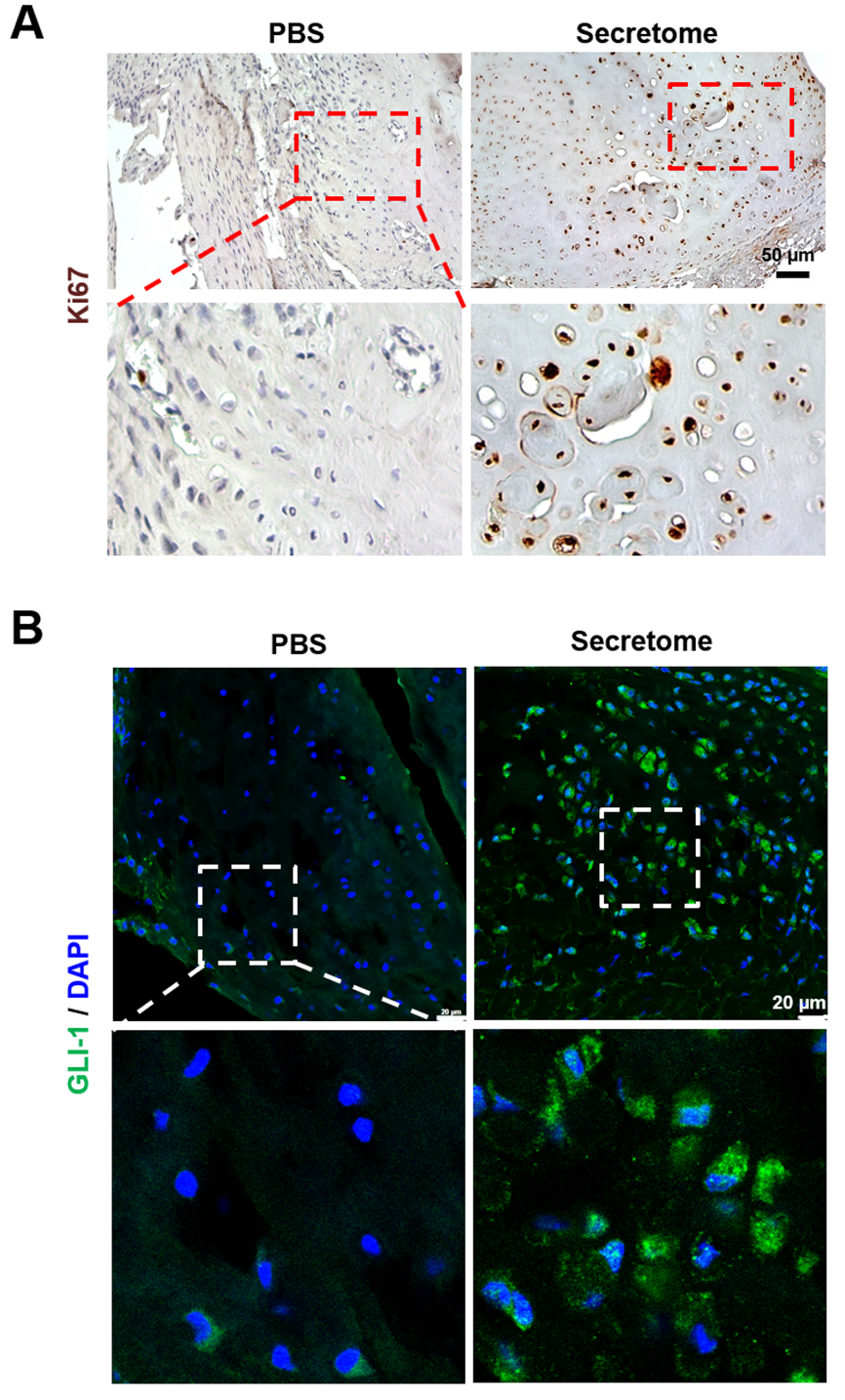


**Supplementary Figure S2. Mouse meniscus tissue after 12-week treatment with PBS or the secretome.** **(Related to main Figure 5.)**

The results of immunohistochemical staining with antibodies against Ki67 (A) and Gli-1 (B) are presented.


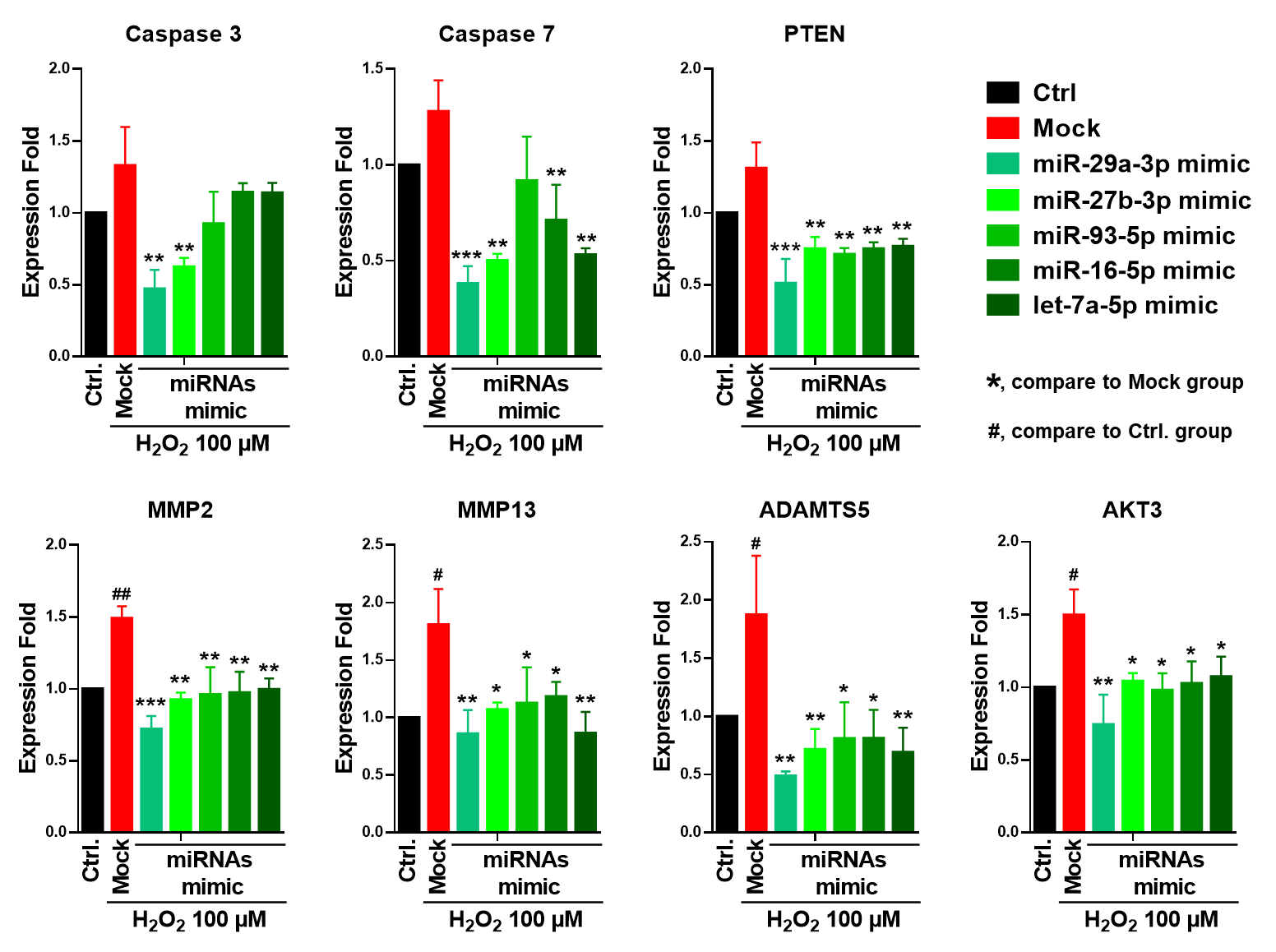


**Supplementary Figure S3. Specific miRNAs repair the injured-biological functions of mouse meniscus progenitor cell. (Related to main Figure 6.)**

Gene expression levels were analyzed by RT-qPCR. For all quantiﬁcations, data are the means ± SEM of at least three independent experiments. ^*^*P* < 0.05, ^**^*P* < 0.01, and ^***^*P* < 0.001; ^#^*P* < 0.05, ^##^*P* < 0.01 (one-way analysis of variance with Tukey’s post hoc test).

**Supplementary** **Table S1.** Antibodies used in this study.

| Protein | Assay | Catalog number | Company | Origin | Dilution | Incubation duration, temperature |
| --- | --- | --- | --- | --- | --- | --- |
| Gli-1 | ICC/ IHC | sc-515751 | Santa Cruz | Mouse | 1:100 | Overnight, 4°C |
| Ki67 | ICC/ IHC | ab15580 | Abcam | Rabbit | 1:100 | Overnight, 4°C |
| TNFα | IHC | sc-133192 | Santa Cruz | Mouse | 1:100 | Overnight, 4°C |
| Sca-1 (Ly-6A/E) | ICC | sc-52601 | Santa Cruz | Rat | 1:100 | Overnight, 4°C |
| Stro-1 | ICC | sc-47733 | Santa Cruz | Mouse | 1:100 | Overnight, 4°C |
| CD44 | ICC/FCM | 12-0441-81 | eBioscience | Rat | 1:50 | Overnight, 4°C |
| CD34 | ICC | 551387 | BD Pharmingen | Rat | 1:50 | Overnight, 4°C |
| Cleaved caspase-3 | ICC | #9661 | Cell Signaling Technology | Rabbit | 1:100 | Overnight, 4°C |
| Sca-1 | FCM | 565355 | BD Biosciences | Rat | 1:20 | Overnight, 4°C |
| CD105 | FCM | 12–1057-82 | Thermo Fisher Scientific | Rat | 1:200 | Overnight, −20°C |
| CD140 | FCM | 13-1401-80 | eBioscience | Rat | 1:200 | Overnight, −20°C |
| CD34 | FCM | 152217 | BioLegend | Rat | 1:200 | Overnight, 4°C |

**Abbreviations:** ICC, immunocytochemistry; IHC, immunohistochemistry; FCM, flow cytometry.

**Supplementary** **Table S2.** Real-time qPCR primers used in this study

| Gene | Species | Accession | Primer | Sequence | Product Size (bp) |
| --- | --- | --- | --- | --- | --- |
| *Caspase 3* | Mouse | NM_001284409 | Forward  Reverse | GAGCTTGGAACGGTACGCTA  GAGTCCACTGACTTGCTCCC | 118 |
| *Caspase 7* | Mouse | NM_007611 | Forward  Reverse | CCGTCCACAATGACTGCTCTTG  CCCGTAAATCAGGTCCTCTTCC | 131 |
| Mmp2 | Mouse | NM_008610 | Forward  Reverse | CAAGGATGGACTCCTGGCACAT  TACTCGCCATCAGCGTTCCCAT | 138 |
| Mmp13 | Mouse | NM_008607 | Forward  Reverse | GACCCCAACCCTAAGCATCC  CCTCGGAGACTGGTAATGGC | 80 |
| Pten | Mouse | NM_008960 | Forward  Reverse | TGAGTTCCCTCAGCCATTGCCT  GAGGTTTCCTCTGGTCCTGGTA | 138 |
| Adamts5 | Mouse | NM_011782 | Forward  Reverse | AGGCACTTGTATGGTTTCTGT  ACCAAACTATTCGGTTAGGCTGA | 98 |
| Akt3 | Mouse | NM_011785 | Forward  Reverse | GAGATGGATGCGTCTACAACCC  TCCACTTGCCTTCTCTCGAACC | 120 |
| β2m | Mouse | NM_009735 | Forward  Reverse | ACAGTTCCACCCGCCTCACATT  TAGAAAGACCAGTCCTTGCTGAAG | 105 |
